# Supplementary material for: Balanced gene dosage control rather than parental origin underpins genomic imprinting
Source: Nat Commun. 2022 Jul 29;13:4391. doi: 10.1038/s41467-022-32144-z (PMC9338321; doi:10.1038/s41467-022-32144-z)
Supplement: Supplementary file 1 — Supplementary Information [file 41467_2022_32144_MOESM1_ESM.pdf]

SUPPLEMENTAL INFORMATION FOR:

BALANCED GENE DOSAGE CONTROL RATHER THAN PARENTAL ORIGIN UNDERPINS GENOMIC IMPRINTING

Ariella Weinberg-Shukron[1,2,3], Raz Ben-Yair[1,3], Nozomi Takahashi[2], Marko Dunjić[1], Alon Shtrikman[1], Carol Edwards[2], Anne C. Ferguson-Smith[2,#], and Yonatan Stelzer[1,#]

[1] Department of Molecular Cell Biology, Weizmann Institute of Science, 7610001 Rehovot, Israel

[2] Department of Genetics, University of Cambridge, Cambridge CB2 3EH, United Kingdom

[3] These authors contributed equally

[#] Co-corresponding authors: afsmith@gen.cam.ac.uk (A.F.S.), yonatan.stelzer@weizmann.ac.il (Y.S.)

Supplementary Table 1- gRNAs and homology arms for targeting and cloning:

Primer	Sequence 5’ → 3’
IG-CGI gRNA-1, Forward	CACCgTACACGCTATATTTGTGCTA
IG-CGI gRNA-1, Reverse	AAACTAGCACAAATATAGCGTGTAc
IG-CGI gRNA-2, Forward	CACCgTACAGACTGTAGTTTAGCTT
IG-CGI gRNA-2, Reverse	AAACAAGCTAAACTACAGTCTGTAc
5’ Arm SBFI, Forward	AACTGAcctgcaggGACTGAATGTTCTTGTGGCAAAGG
5’ Arm Bamh1/Loxp/Nhe1, Reverse	AACTGAgctagcataacttcgtataatgtatgctatacgaagttatggatccGCGTGTACTATAAAATGGCTGCATG
IG-CGI Nhe1, Forward	AACTGAgctagcGTGCTAAGGTACATCATGCTAGTG
IG-CGI HindIII/LoxP/Pac1, Reverse	AACTGAttaattaaataacttcgtataatgtatgctatacgaagttataagcttACTACAGTCTGTATGGTCACAGC
3’ Arm PacI, Forward	AACTGAttaattaaGCTTTGGAATTCCTGATGGAATCTG
3’ Arm FseI, Reverse	AACTGAggccggccCATCCCACGAGGAACTGGTAAAC

Supplementary Table 2- genotyping primers:

Primer	Sequence 5’ → 3’
IG-CGI 5’SQ Genotyping, Forward	AGCAAACCTTCACTAGGGAACAGG
IG-CGI 5’SQ Genotyping, Reverse	TGCCCATGACAAACCACAGC
IG-CGI 5’SQ Genotyping Unique, Reverse	cgaagttatggatccGCGTGTAC
IG-CGI 3’SQ Genotyping, Forward	ATTGGGAATGGGATCACGCG
IG-CGI 3’SQ Genotyping, Reverse	AAGCAATCCCACCACAGCTTC
IG-CGI 3’SQ Genotyping Unique, Forward	CCATACAGACTGTAGTaagcttataacttcg
IG-CGI loxP Genotyping, Forward	GTCTATATGGCACCATGCAGCC
IG-CGI loxP Genotyping, Reverse	CTCTGCAAGTACCAGATTCCATCAG
IG-CGI$^{f/f}$ Forward	GTCTATATGGCACCATGCAGCC
IG-CGI$^{f/f}$ Reverse	CTCTGCAAGTACCAGATTCCATCAG
IG-CGI$^{f/f}$ Internal Reverse	CTCGCTAGTTCACGGAGGTC
Gtl2-DMR Genotyping, Forward	GTAACGGGCAGAAATGGGTC
Gtl2-DMR Genotyping, Reverse	CCCACACCCGCGAGAAAA

Gtl2-DMR Internal Reverse	ACTGCAGCAAAGAACGAAGG
Rosa26:lsl-tdTomato, WT, Forward	AAGGGAGCTGCAGTGGAGTA
Rosa26:lsl-tdTomato, WT, Reverse	CCGAAAATCTGTGGGAAGTC
Rosa26:lsl-tdTomato, Transgene, Forward	GGCATTAAAGCAGCGTATCC
Rosa26:lsl-tdTomato, Transgene, Reverse	CTGTTCCTGTACGGCATGG
Vasa-Cre, Transgene, Forward	CACGTGCAGCCGTTTAAGCCGCGT
Vasa-Cre, Transgene, Reverse	TTCCCATTCTAAACAACACCCTGAA

Supplementary Table 3- PCR primers for Bisulfite Sequencing:

Primer	Sequence 5' → 3'
IG-CGI Nested, Forward	GGTATATTATGTTAGTGTTAGGAAGGATTGTG
IG-CGI Nested, Reverse	CATCCCCTATACTCAAAACATTCTCC
IG-CGI Internal, Forward	AAGTGTTGTGGTTTGTTATGGGTAAG
IG-CGI Internal, Reverse	ATAATACAACCCTTCCCTCACTCC
Gtl2-DMR Nested, Forward	GGGTATTTTATGTGGGGGTGATAG
Gtl2-DMR Nested, Reverse	TCAAACAAAAAATAACTAACCACTCACC
Gtl2-DMR Internal, Forward	GGGTGATAGTTTTTAGGTTAATATTTGGG
Gtl2-DMR Internal, Reverse	AACTACAACCAAAACCAACAAAACC
IG-CGI pyro, Forward	GTGGTTTGTTATGGGTAAGTTT
IG-CGI pyro, Reverse	CCCTTCCCTCACTCCAAAAATTAA
IG-CGI pyro, Sequencing	TGGTTTATTGTATATAATGT
IG-TRE pyro, Forward	GTTGGGGTTTGTTAGTTATTTATATGTTAT
IG-TRE pyro, Reverse	AAAACATACTCTCCACTATAACTAATT
IG-TRE pyro, Sequencing	CTATAACTAATTACAACACCAC
Gtl2-DMR pyro, Forward	AGTTATTTTTTGTTTGAAAGGATGTGTA
Gtl2-DMR pyro, Reverse	CTAACTTTAAAAAAAAATCCCCAACACT
Gtl2-DMR pyro, Sequencing	GAAAGGATGTGTAAAAATGA

Supplemental Table 4- quantitative real-time PCR primers:

Primer	Sequence 5’ → 3’
GAPDH Forward	AGGTCGGTGTGAACGGATTTG
GAPDH Reverse	TGTAGACCATGTAGTTGAGGTCA
β-Actin Forward	GGC TGT ATT CCC CTC CAT CG
β-Actin Reverse	CCA GTT GGT AAC AAT GCC ATG T
Dlk1 Forward	ACTTGCGTGGACCTGGAGAA
Dlk1 Reverse	CTGTTGGTTGCGGCTACGAT
Gtl2 Forward	TCCTCACCTCCAATTTCCCCT
Gtl2 Reverse	GAGCGAGAGCCGTTCGATG
Rian Forward	TCGAGACACAAGAGGACTGC
Rian Reverse	TCGCATCAAGCTTAGACCTG
Mirg Forward	GACTTTGTGGCCTCCCTTC
Mirg Reverse	GGAGCATCTTCTGGAGTCAAA
Dio3 Forward	CACGGCCTTCATGCTCTGG
Dio3 Reverse	CGGTTGTCGTCTGATACGCA

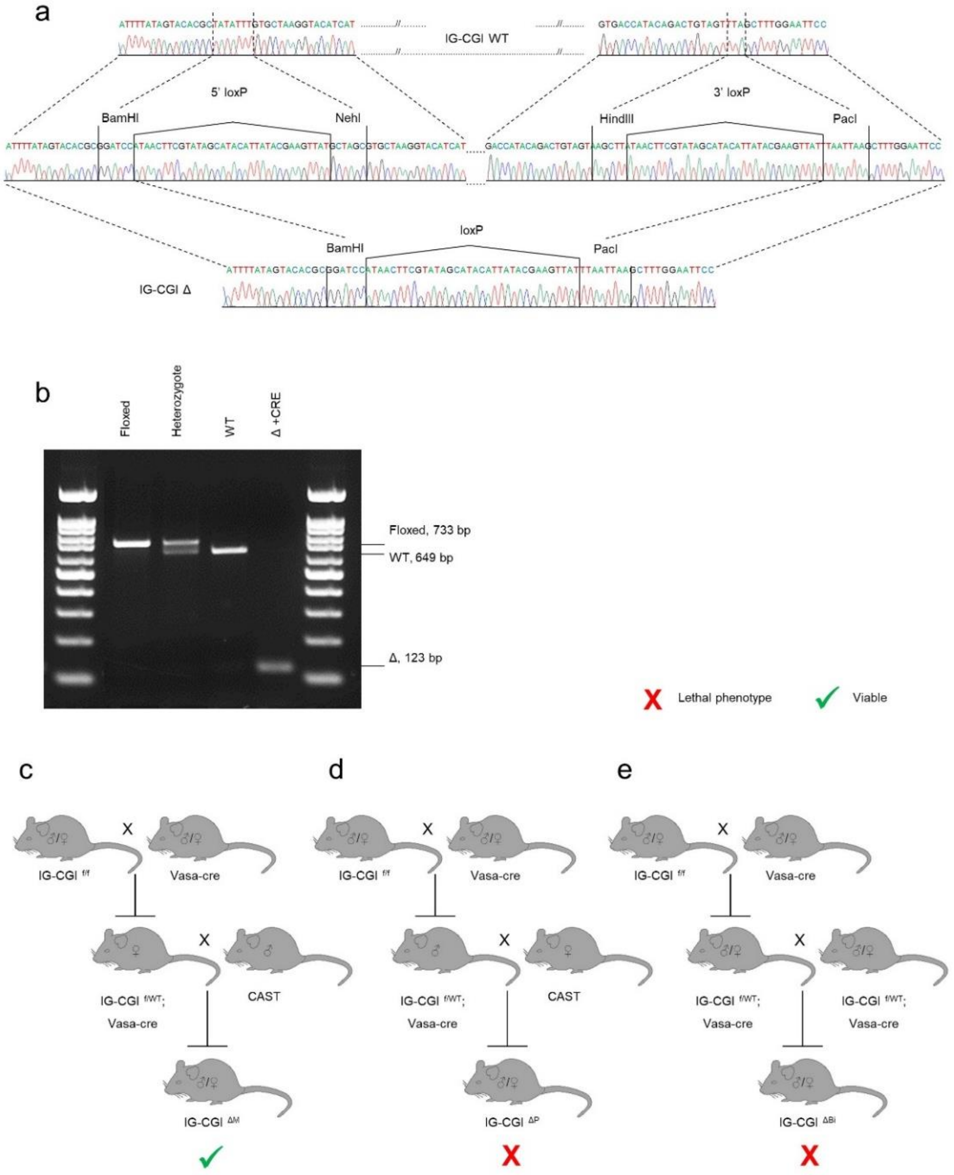

Supplementary Figure 1. A transgenic system for germline deletion of the IG-CGI. a, Schematic representation of Cre-lox mediated targeting of the IG-CGI in mouse embryonic stem cells. **Top row**: WT sequence. **Middle row**: insertion of loxP sites flanking the IG-CGI. **Bottom row**: Cre-induced deletion of the IG-CGI retains a single loxP site. **b**, PCR result for the different genotype primers presented in Fig. 1a. Respective sizes are depicted on the right. Results are shown for a representative cell clone and were repeated in 6 paternal floxed and 4 maternal floxed mESCs with and without CRE. **c**-**e,** Schemes showing the mating strategy for *in-vivo* deletion of the IG-CGI. F1 mice (second row) harbor the IG-CGI deletion in their germ cells. F2 mice (third row) carry the deletion ubiquitously in a parent specific manner. **c,** Maternal IG-CGI deletion. **d,** Paternal IG-CGI deletion. **e,** Biallelic IG-CGI deletion.

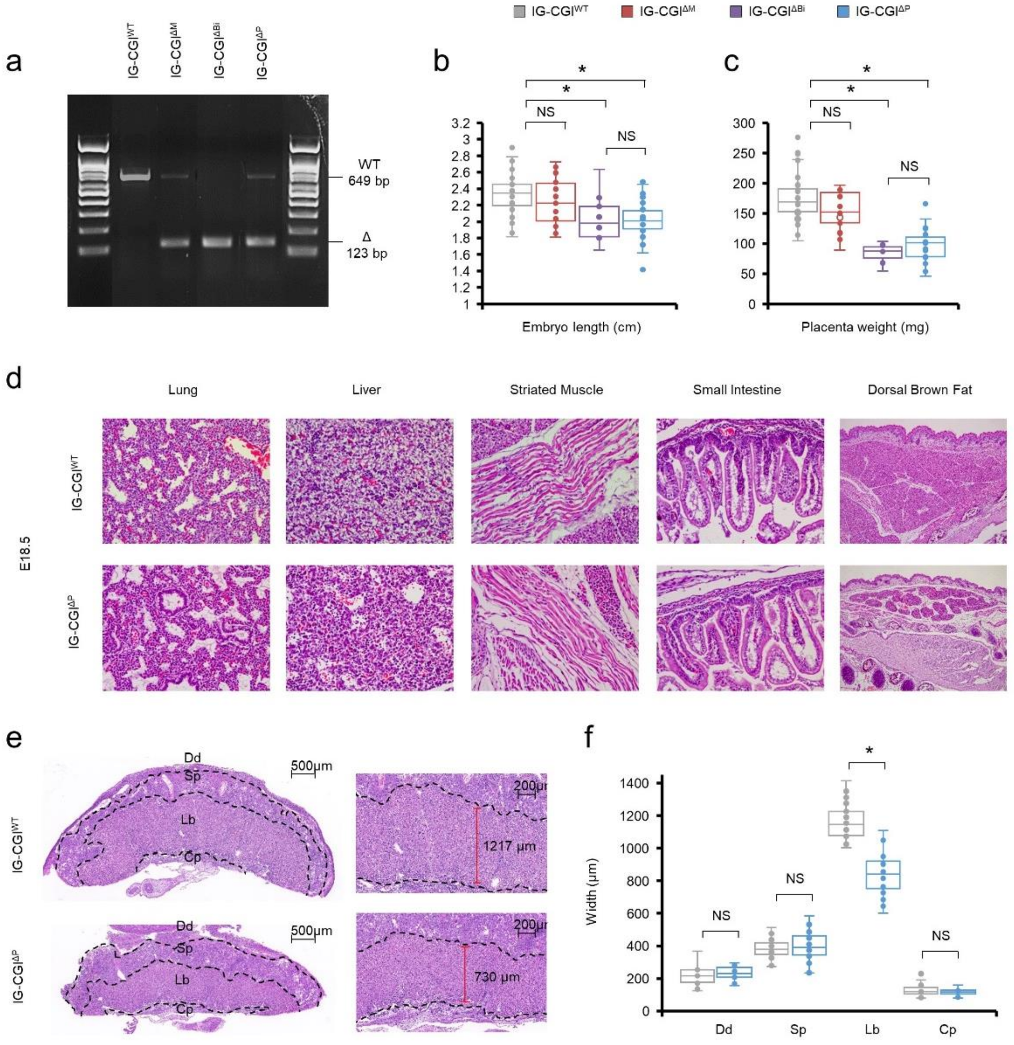

g

Numbers and survival rates in germline IG-CGI parent-of-origin deletion embryos

Transmission	Genotype	E8.5	E18.5	P21 (3 weeks)
WT	IG-CGI^{WT}	25	77	77
Maternal	IG-CGI$^{\Delta M}$; Vasa-Cre	17	32	25
Biallelic	IG-CGI$^{\Delta Bi}$; Vasa-Cre	3	15(3)	2(15) *
Paternal	IG-CGI$^{\Delta P}$; Vasa-Cre	13	70(3)	4(55) *

Supplementary Figure 2. Germline paternal deletion of the IG-CGI leads to small embryos with deficient placentas. a, PCR result for the different genotype primers presented in Fig. 1a. Respective sizes are depicted on the right. Results are shown for one embryo from each genotype but were repeated independently for all embryos analyzed, as described in panel **(g)** (179 WT embryos, 74 ΔM embryos, 38 ΔBi embryos and 145 ΔP embryos). **b**, Box-plot showing distribution of embryo head-to-tail lengths per genotype group. N_{WT}=72, $N_{\Delta M}$=27, $N_{\Delta Bi}$=16, $N_{\Delta P}$=62 biologically independent embryos. Box plot minima= 1.82, 1.81, 0.67, 1.42; maxima=

2.9, 2.73, 2.64, 2.48; center= 2.35, 2.22, 1.98, 2. For WT, ΔM, ΔBi and ΔP respectively. Bounds of boxes show the 25th and 75th percentiles. Whiskers extend 1.5 times the interquartile range. NS- not significant. Asterisks indicate statistical significance in comparison to WT using a one-way ANOVA (ΔBi: p= 2.8e-6, ΔP: p= 1.3e-14). **c**, Box-plot representing placenta weight distribution for the different genotype groups from the corresponding embryos in (**b**). Box plot minima= 92.4, 89.2, 54.3, 45.7; maxima= 276.2, 196.9, 103.7, 174; center= 164.8, 152.4, 87.8, 100.9. For WT, ΔM, ΔBi and ΔP respectively. Bounds of boxes show the 25th and 75th percentiles. Whiskers extend 1.5 times the interquartile range. NS- not significant. Asterisks indicate statistical significance in comparison to WT using a one-way ANOVA (ΔBi: p= 2e-13, ΔP: p= 1.37e-24). **d**, Representative H&E–stained histological sections of various tissues from one E18.5 IG-CGI$^{\Delta P}$ and one WT littermate control out of 4 biologically independent embryos that were analyzed. **e**, Representative H&E-stained histological sections of placentas from E18.5 embryos. N=4 biologically independent tissues for both WT and IG-CGI$^{\Delta P}$ embryos. Left: Transverse H&E-stained sections. Dd- Decidua; Sp- Spongiotrophoblast (junctional zone); Lb- Labyrinth; Cp- Chorionic plate. Right: enlarged insets showing labyrinth zone. **f**, Box-plot showing the distribution of placenta layer widths in the IG-CGI$^{\Delta P}$ and WT control. N=40 biologically independent tissues per group. Dd Box plot minima= 126.1, 156.9; maxima= 366, 296.5; center= 216.4, 230.1. Sp Box plot minima= 273, 233,6; maxima= 512.6, 585.3; center= 377.8, 389.5. Lb Box plot minima= 1002.5, 600; maxima= 1413.8, 1109.6; center= 1144.8, 840.4. Cp Box plot minima= 79.1, 78.6; maxima= 229.3, 159; center= 120, 117.4. For WT and ΔP respectively. Bounds of boxes show the 25th and 75th percentiles. Whiskers extend 1.5 times the interquartile range. NS- not significant. Asterisks indicate statistical significance in comparison to WT using a one-way ANOVA (p= 6.66e-22). **g,** Embryo numbers and survival rates in germline IG-CGI deletion embryos. Numbers in parentheses represent dead embryos/pups. Asterisks indicate a statistically significant difference between the expected and observed genotype frequencies using a Chi-squared test (ΔBi p= 4.11e-6, ΔP p= 5.99e-6).

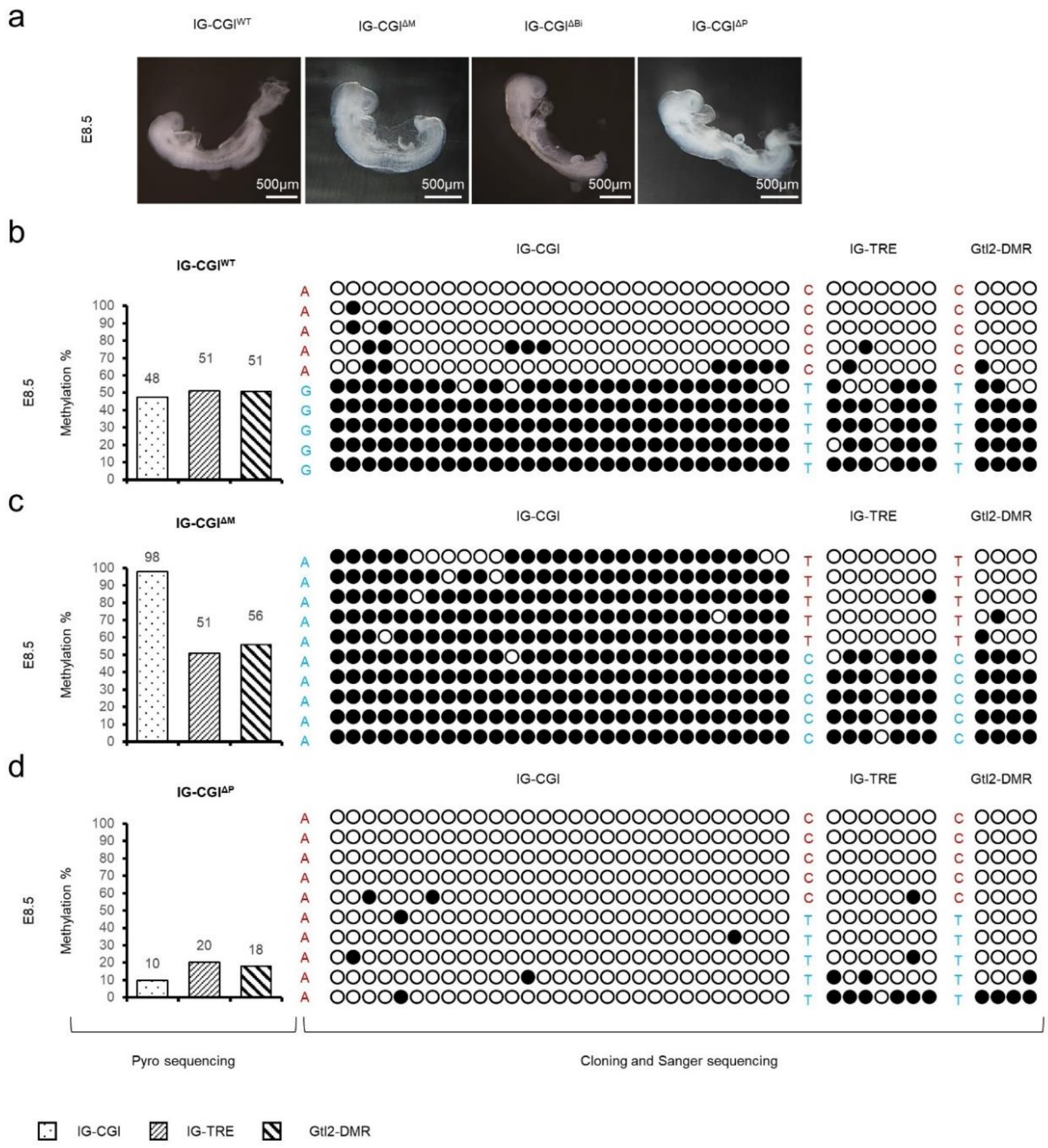

Supplementary Figure 3. Allele-specific methylation analysis in E8.5 embryos. a, Representative images of E8.5 embryos from the different genotypes. Mutant embryos are indistinguishable from their WT littermates, with clear headfolds, heart rudiment and first somites. **b-d**, Analysis of methylation levels using bisulfite sequencing on regulatory elements of the Dlk1-Dio3 locus with allele-specific resolution. Each row represents a PCR amplicon followed by Sanger sequencing. Open circles represent unmethylated CpGs; black circles - methylated CpGs. Summaries of methylation levels for each region is presented in bar graphs on the left. Letters represent SNPs from the maternal (red) and paternal (blue) alleles discriminated by crossing BL6 and CAST strains. **b,** Wild-type embryos. **c,** Maternal IG-CGI deletion. **d,** Paternal IG-CGI deletion.

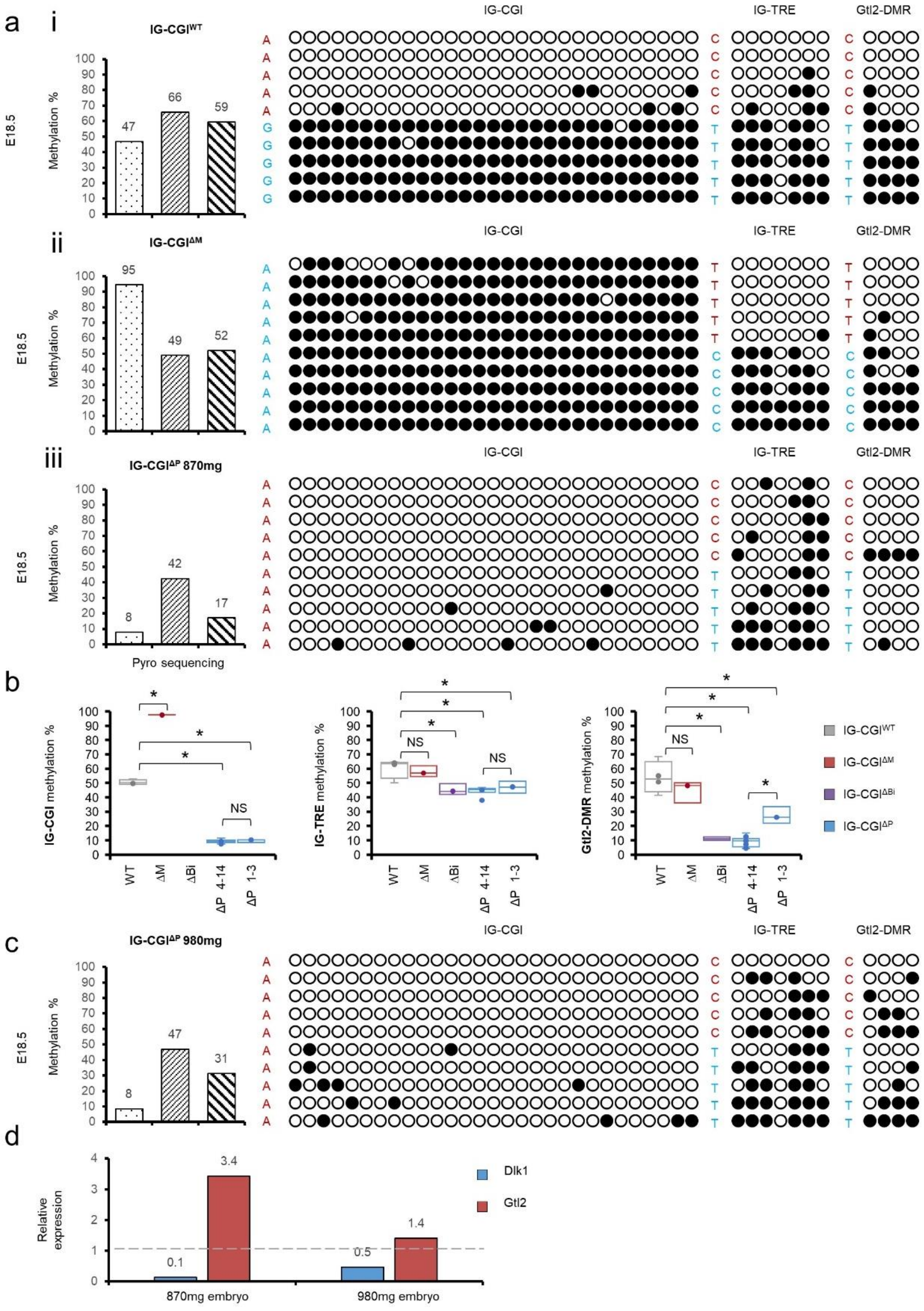

Supplementary Figure 4. Allele-specific methylation analysis in E18.5 embryos. a, Analysis of methylation levels using bisulfite sequencing on regulatory elements of the Dlk1-Dio3 locus with allele-specific resolution. Each row represents a PCR amplicon followed by Sanger sequencing. Open circles represent unmethylated CpGs; black circles - methylated CpGs.

Summaries of methylation levels for each region is presented in bar graphs on the left. Letters represent SNPs from the maternal (red) and paternal (blue) alleles discriminated by crossing BL6 and CAST strains. **b**, Box plots representing association between genotypes and methylation levels on IG-CGI (left), IG-TRE (middle) or Gtl2-DMR (right). IG-CGI$^{\Delta P}$ embryos 4-14 are typically underweight while IG-CGI$^{\Delta P}$ embryos 1-3 are larger, as described in Fig. 1i, j. IG-CGI Box plot minima= 49.1, 97.5, -, 7.3, 8.5; maxima= 52.7, 97.7, -, 11.5, 10.4; center= 50.3, 97.5, -, 9.4, 10.2. IG-TRE Box plot minima= 62.9, 56.9, 42, 37.9, 43.1; maxima= 66.1, 65, 49.4, 46.9, 51.4; center= 64.3, 62.1, 44.2, 45.2, 47.2. Gtl2-DMR Box plot minima= 41.5, 36.1, 10, 4.2, 22; maxima= 68.4, 50, 12.3, 15, 33.4; center= 52.8, 48.2, 11.1, 9.6, 26. For WT, ΔM, ΔBi, ΔP embryos 4-14 and ΔP embryos 1-3 respectively. Bounds of boxes show the 25th and 75th percentiles. Whiskers extend 1.5 times the interquartile range. NS- not significant. Asterisks indicate statistical significance in comparison to WT using a one-way ANOVA (IG-CGI: p= 6.1e-8, 1.3e-14, and 0.05 for ΔM, ΔP embryos 4-14 and ΔP embryos 1-3 respectively. IG-TRE: p= 0.0002, 8.4e-9 and 0.0005 for for ΔBi, ΔP embryos 4-14 and ΔP embryos 1-3 respectively. Gtl2-DMR: p= 0.007, 1.3e-8 and 0.01 for Bi, ΔP embryos 4-14 and ΔP embryos 1-3 respectively. p= 1.2e-05 for Gtl2-DMR between ΔP embryos 4-14 and 1-3). **c**, Methylation analysis in an atypical IG-CGI$^{\Delta P}$ embryo that attained normal weight at E18. **d**, Bar graph representing qRT-PCR relative expression values of *Dlk1* and *Gtl2* in the typical and atypical IG-CGI$^{\Delta P}$ embryos analyzed in a and c.

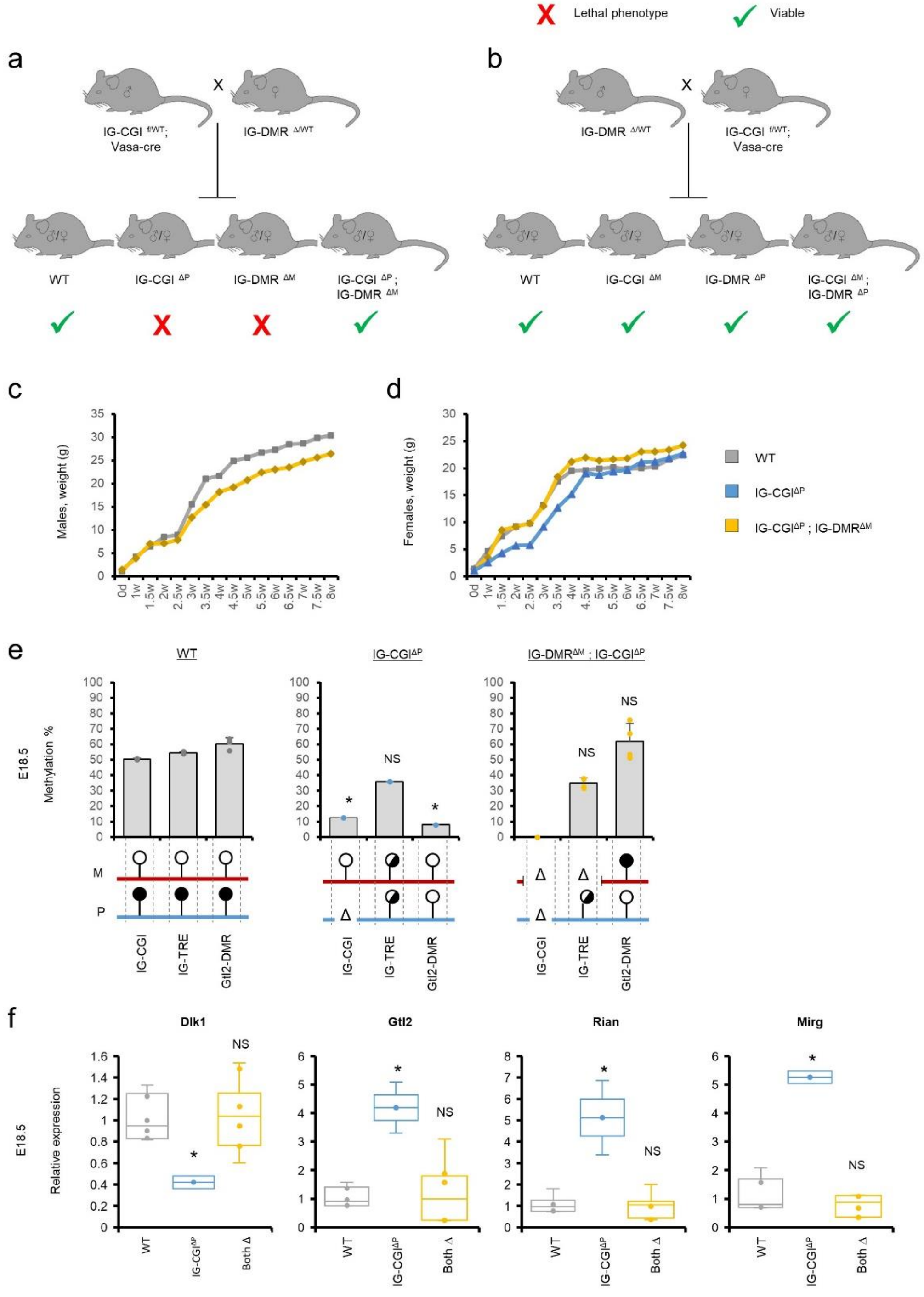

Supplementary Figure 5. Mice carrying paternal IG-CGI and maternal IG-DMR deletion exhibit inverted imprinting, postnatal viability, and long term survival. a, b, Scheme demonstrating mating strategy for generation of double deletion mice. **c-d**, Plots showing pup weight gain in males (**c**) and females (**d**). N_{WT}=6,2 and $N_{\Delta P}$=5,2 biologically independent animals for male and female pups, respectively. **e**, Bar-graphs showing methylation levels analyzed using bisulfite pyro-sequencing, on regulatory elements of the Dlk1-Dio3 locus in E18.5 embryos of

different genotype groups. Schemes below graphs depict inferred allele-specific methylation status. N(WT)=6, N(IG-CGI$^{\Delta P}$)=3, N(IG-DMR$^{\Delta M}$; IG-CGI$^{\Delta P}$)=8 biologically independent embryos. Data are presented as mean values +/- SD. NS- not significant. Asterisks indicate statistical significance in comparison to WT using a one-way ANOVA (IG-CGI$^{\Delta P}$: p= 0.0001 and 0.007 for IG-CGI and Gtl2-DMR respectively). **f**, Box-plots representing qRT-PCR analysis of representative genes in the Dlk1-Dio3 region in E18.5 embryos of different genotype groups. N(WT)=3, N(IG-CGI$^{\Delta P}$)=2, N(IG-DMR$^{\Delta M}$; IG-CGI$^{\Delta P}$)=4 biologically independent embryos. Dlk1 Box plot minima= 0.8, 0.4, 0.6; maxima= 1.3, 0.5, 1.5; center= 0.9, 0.4, 1. Gtl2 Box plot minima= 0.8, 3.3, 0.2; maxima= 1.6, 5.1, 3.1; center= 0.9, 4.2, 1. Rian Box plot minima= 0.7, 3.4, 0.3; maxima= 1.8, 6.9, 2; center= 1, 5.1, 1. Mirg Box plot minima= 0.7, 5.1, 0.3; maxima= 2.1, 5.5, 1.1; center= 0.8, 5.3, 0.9. For WT, IG-CGI$^{\Delta P}$ and IG-DMR$^{\Delta M}$;IG-CGI$^{\Delta P}$ respectively. Bounds of boxes show the 25th and 75th percentiles. Whiskers extend 1.5 times the interquartile range. NS- not significant. Asterisks indicate statistical significance in comparison to WT using a one-way ANOVA (p= 0.002, 9.14e-5, 0.0006 and 7.57e-6 for Dlk1, Gtl2, Rian and Mirg respectively).
