# Supplementary material for: Balanced gene dosage control rather than parental origin underpins genomic imprinting
Source: Nat Commun. 2022 Jul 29;13:4391. doi: 10.1038/s41467-022-32144-z (PMC9338321; doi:10.1038/s41467-022-32144-z)
Supplement: Supplementary file 3 — Reporting Summary [file 41467_2022_32144_MOESM3_ESM.pdf]

nature portfolio

Corresponding author(s): Dr. Yonatan Stelzer

Last updated by author(s): Jul 7, 2022

Reporting Summary

Nature Portfolio wishes to improve the reproducibility of the work that we publish. This form provides structure for consistency and transparency in reporting. For further information on Nature Portfolio policies, see our Editorial Policies and the Editorial Policy Checklist.

Statistics

For all statistical analyses, confirm that the following items are present in the figure legend, table legend, main text, or Methods section.

n/a	Confirmed	
☐	☒	The exact sample size (*n*) for each experimental group/condition, given as a discrete number and unit of measurement
☐	☒	A statement on whether measurements were taken from distinct samples or whether the same sample was measured repeatedly
☐	☒	The statistical test(s) used AND whether they are one- or two-sided<br>*Only common tests should be described solely by name; describe more complex techniques in the Methods section.*
☐	☒	A description of all covariates tested
☐	☒	A description of any assumptions or corrections, such as tests of normality and adjustment for multiple comparisons
☐	☒	A full description of the statistical parameters including central tendency (e.g. means) or other basic estimates (e.g. regression coefficient) AND variation (e.g. standard deviation) or associated estimates of uncertainty (e.g. confidence intervals)
☐	☒	For null hypothesis testing, the test statistic (e.g. *F*, *t*, *r*) with confidence intervals, effect sizes, degrees of freedom and *P* value noted<br>*Give P values as exact values whenever suitable.*
☒	☐	For Bayesian analysis, information on the choice of priors and Markov chain Monte Carlo settings
☒	☐	For hierarchical and complex designs, identification of the appropriate level for tests and full reporting of outcomes
☐	☒	Estimates of effect sizes (e.g. Cohen's *d*, Pearson's *r*), indicating how they were calculated

Our web collection on statistics for biologists contains articles on many of the points above.

Software and code

Policy information about availability of computer code

Data collection	No software was used for data collection
Data analysis	Embryo images were captured on a Nikon SMZ18 Stereo Microscope and processed with NIS-Elements D Imaging Software (Nikon), ImageJ, and Adobe Photoshop. Histological sections were scanned on the 3D Histech Pannoramic midi camera and analyzed using the CaseViewer Digital Slide Viewer.

For manuscripts utilizing custom algorithms or software that are central to the research but not yet described in published literature, software must be made available to editors and reviewers. We strongly encourage code deposition in a community repository (e.g. GitHub). See the Nature Portfolio guidelines for submitting code & software for further information.

Data

Policy information about availability of data

All manuscripts must include a data availability statement. This statement should provide the following information, where applicable:

- Accession codes, unique identifiers, or web links for publicly available datasets
- A description of any restrictions on data availability
- For clinical datasets or third party data, please ensure that the statement adheres to our policy

Source data are provided with this paper. The PBAT data discussed in this publication have been deposited in NCBI's Gene Expression Omnibus (Edgar et al., 2002) and are accessible through GEO Series accession number GSE207600 (https://www.ncbi.nlm.nih.gov/geo/query/acc.cgi?acc=GSE207600).

Human research participants

Policy information about studies involving human research participants and Sex and Gender in Research.

Reporting on sex and gender	not applicable
Population characteristics	not applicable
Recruitment	not applicable
Ethics oversight	not applicable

Note that full information on the approval of the study protocol must also be provided in the manuscript.

Field-specific reporting

Please select the one below that is the best fit for your research. If you are not sure, read the appropriate sections before making your selection.

☒ Life sciences ☐ Behavioural & social sciences ☐ Ecological, evolutionary & environmental sciences

For a reference copy of the document with all sections, see nature.com/documents/nr-reporting-summary-flat.pdf

Life sciences study design

All studies must disclose on these points even when the disclosure is negative.

Sample size	No sample size calculation was performed. At least three biological replicates were used for each measurement from at least three different litters. Each litter has a mix of deletion and WT littermates. To minimize litter specific effects we pooled embryos from the same genotype from several litters, and at least more than three since three biological independent replicates is the minimum needed for statistical significance. For most analysis three was a minimum as the more samples tested the stronger the significance is. We therefore sampled enough embryos to obtain statistically significant results, without wasting to many animals (according to the Reduction ethical use of animal models).
Data exclusions	No data were excluded from the analysis
Replication	Results were replicated by analysis of embryos and pups from multiple litters to minimize litter specific effects and maximize reproducibility of data. 17 independent litters were analyzed in the course of three months on a B6 mouse background and then repeated in crosses with CAST mice. All attempts at replication were successful.
Randomization	Embryos were genotyped by PCR and allocated into experimental groups according to their genotype
Blinding	All measurements of embryo/pups were divided into groups by genotypes. Blinding was not possible because we needed to know which embryos were from the same genotype to analyze together. In addition, from E18.5 onwards the phenotype was very apparent with paternal deletion embryos dying after birth, hence it could not be possible to blind the allocation of genotypes. When analyzing images of embryos/ tissues to determine histological differences blind analysis was performed by a third party investigator who did not know the genotype of the samples.

Reporting for specific materials, systems and methods

We require information from authors about some types of materials, experimental systems and methods used in many studies. Here, indicate whether each material, system or method listed is relevant to your study. If you are not sure if a list item applies to your research, read the appropriate section before selecting a response.

Materials & experimental systems

n/a	Involved in the study	
☒	☐	Antibodies
☐	☒	Eukaryotic cell lines
☒	☐	Palaeontology and archaeology
☐	☒	Animals and other organisms
☒	☐	Clinical data
☒	☐	Dual use research of concern

Methods

n/a	Involved in the study	
☒	☐	ChIP-seq
☒	☐	Flow cytometry
☒	☐	MRI-based neuroimaging

Eukaryotic cell lines

Policy information about cell lines and Sex and Gender in Research

Cell line source(s)	Mouse embryonic stem cells (Jaenisch lab, MIT. RRID:CVCL_C865)
Authentication	Karyotyping, PCR and Sanger Sequencing
Mycoplasma contamination	All cell lines were tested negative for mycroplasma contamination
Commonly misidentified lines (See ICLAC register)	No commonly misidentified cell lines were used in the study

Animals and other research organisms

Policy information about studies involving animals; ARRIVE guidelines recommended for reporting animal research, and Sex and Gender in Research

Laboratory animals	Blastocyst injections were performed in BDF2 diploid blastocysts, harvested from hormone primed BDF1 4 week-old females. For germline transmission, male chimera mice were mated to 6-8 week old C57BL/6 females. Female and male Vasa-Cre mice (FVB-Tg(Ddx4-cre)1Dcas/J) Jackson Laboratory stock#006954) were used to create maternal or paternal deletion in the germcells, respectively. Female and male C57BL/6 or CAST/EiJ (RRID:IMSR_JAX:000928) mice were used for pure and hybrid breeding experiments. Mice were mated at 8–12 weeks of age. F2 embryos harboring the deletion allele were analyzed at different ages: E8.5, E18.5, postnatal day 1 (P1) or post-weaning (P21).<br>We also added the following information in the manuscript, regarding housing conditions for the mice: "mice were maintained under a 12 hr light–dark cycle at 22°C degrees (+/- 1°C) and 50% humidity (+/-10%). Mice were monitored for health and activity and were given ad libitum access to water and standard mouse chow".
Wild animals	No wild animals were used in the study
Reporting on sex	Embryos were assayed for sex by PCR for the SRY gene. Pups were sexed at weaning. no sex bias was observed between genotypes.
Field-collected samples	No field collected samples were used in the study
Ethics oversight	All animal experiments were performed according to the Animal Protection Guidelines of Weizmann Institute of Science, Rehovot, Israel and in accordance with the Animals (Scientific Procedures) Act 1986 Amendment Regulations 2012 following ethical review by the University of Cambridge Animal Welfare and Ethical Review Body. Animal experiments were approved by relevant Weizmann Institute IACUC (#39401117-3 and #00080118-2) and UK Home Office project license #PC213320E. All efforts were made to minimize animal discomfort.

Note that full information on the approval of the study protocol must also be provided in the manuscript.
